# Automated software bug severity classification using ensemble machine learning scheme: A real case study

**DOI:** 10.1371/journal.pone.0330510

**Published:** 2025-10-15

**Authors:** Mohammadreza Namdar, Farnaz Barzinpour, Rassoul Noorossana, Mohammad Saidi-Mehrabad

**Affiliations:** 1 School of Industrial Engineering, Iran University of Science and Technology, Tehran, Iran; 2 Information Systems and Operations Management Department, College of Business, University of Central Oklahoma, Edmond, Oklahoma, United States of America; Wilfrid Laurier University - Waterloo Campus: Wilfrid Laurier University, CANADA

## Abstract

Software bug report classification is one of the most significant processes in software development for determining the nature and severity of faults based on their causes and effects. In many projects, software experts implement this process manually, which requires exorbitant time and effort. Although there are a few studies on automatic bug report classification using machine learning techniques, they mainly focus on structured open-source datasets. This paper presents an ensemble learning approach utilizing various multiclass machine learning, text classification, and natural language processing techniques for automated software bug severity classification, with an application in the Persian language. This language, due to its unique characteristics, requires the adoption of different approaches from those applicable to the English language for text classification. The proposed approach utilizes a real bug dataset extracted from a case study containing unstructured bug reports. This dataset contains 4429 bug reports about the software product of the studied company, which is used by thousands of users in government and private organizations. These bug reports were recorded in Persian text by the testing team or software users, and then classified based on their severity through meetings of development team managers in the company. Results demonstrate that the developed appraoch is highly accurate and significantly faster than manual classification, which can dramatically decrease software development time and cost.

## 1. Introduction

Increasing the software projects’ size and complexity has led to more bugs in the development process. This challenge has grown the importance of the software testing process [1]. In the testing process, the textual explanations of discovered bugs are usually described in the bug reports [2]. Due to a large number of bug reports, bug classification is a critical, time-consuming, and tiring challenge for experts. For example, Pingclasai et al. [3] indicated that experts have spent 90 days manually classifying about 7000 bug reports. However, the bug reports classification cannot be ignored because it determines the bug’s priority and severity in terms of its effect on the operational, functional, or security of the software [4]. Also, it decreases the testing process time by improving the bug assignment time to the most appropriate developer. Thus, bug classification using machine learning (ML) methods recently has attracted much attention in this substantial field, which can greatly improve the software systems quality and organizations’ efficiency [5]. Similarly, according to the literatrue, the ML methods have been used to classify various types of texts in different fields, including engineering change documents [6], Arabic articles [7], Chinese news article [8] and etc.

One of the first studies in the field of software bug classification is [9] which suggested a preliminary approach called “Orthogonal Defect Categorization (ODC)” for classifying bugs. Although this paper provided a beneficial framework for software defect classification, conducting the presented model on real bug reports was human-intensive and required experts’ knowledge of both ODC and system domains. Other papers such as [10–11] presented other studies in this field in the following years. In the early 20^th^ century, with the growth of artificial intelligence, many studies such as [12–14] used classifiers based on machine learning, text mining, and Natural language processing (NLP) techniques to automatically categorize bug reports. Although these studies solved some of the weaknesses of traditional research, they only presented classification models on open-source datasets and they were not necessarily suitable for classifying bug reports related to real datasets in case studies. Also, they utilized single classification models, which generally have lower accuracy than ensemble classification models. Moreover, in recent years, applying the NLP and ML concepts in software bug classification has been considered by many authors. For example, Kumar [15] classified bug severity into several categories by using Decision Tree (DT), Naive Bayes (NB), and Bagging. Also, Sarawan et al. [16] have evaluated the efficiency of Logistic Regression (LR), Random Forest (RF), SVM and long-term short-term memory for fault binary classification. Ahmed et al. [17] have presented a framework using NB, RF, DT, and LR to categorize and prioritize bug reports. They was achieved an accuracy of 88.78% by using an RF classifier for predicting the category and an accuracy of 90.43% in predicting the priority of bug reports. Among other articles in this field, can be referred to [18–21].

Although the studies mentioned above have applied ML methods for automated software bug severity classification, they often include several basic disadvantages as follows:

- They often applied different single ML for predicting the class of bug reports while ensemble learning schemes by combining the results of several ML methods can achieve higher classification and recognition performance compared to single models.
- They mostly concentrated on open-source software datasets while software bug classification in actual industrial environments can provide valuable insights into the topic [1].
- They considered English text input and none of them focused on Persian bug reports while the analysis of Persian texts is necessary in different countries which use the Persian language in the software companies. Albeit, it should be noted that a few papers such as [22] utilized ML techniques for sentiment analysis in Persian texts but they are not on the topic of automated software bug classification.
- They usually focus on structured bug reports that contain text and more detailed information about them while unstructured reports classification is complex due to the short text of reports.

To bridge the research gaps in the literature, the proposed paper uses ML techniques such as Support Vector Machines (SVM), Multinomial Naive Bayes (MNB), Gaussian Naive Bayes (GNB), Logistic Regression (LR) and Random Forest (RF) for automated software bug report classification. Next, it combines their predictions of indicated ML algorithms for the final classification of instances using two forms of voting. The classification process will be applied to determine the severity of unstructured bug reports in Persian language text. Due to the particular attributes of the Persian language, the direct use of methods and tools developed for the English language in Persian has its limitations. Hence, it is necessary to present unique approaches for Persian text preprocessing. Moreover, the paper addresses a real case study with a proprietary bug dataset to develop bug classification challenges into business software bug reports. Thus, it provides additional insight into the topic.

Generally, it can be said that the objectives of this study are:

- Presenting an efficient solution based on text mining, ML algorithms, and NLP in the artificial intelligence field to automatically classify software bug severity in the Persian language.
- Comparing the performance of different supervised machine learning algorithms in the bug reports classification based on metrics such as accuracy, precision, recall and f1-score.
- Providing an ensemble learning scheme based on the voting approach to improve the performance and accuracy of single classifiers.

In the continuation of this section, Table 1 provides a summary of state-of-the-art studies in the literature. Then, Section 2 and 3 expresses the problem description and research methodology, respectively. Moreover, Section 4 indicates the results of the model based on a real dataset extracted from an actual case study and compares the performance of algorithms used and ensemble learning scheme. Section 5 and 6 describe discussion and some factors that may threaten the validity of the suggested research, respectively. Finally, Section 7 indicates the conclusions and suggestions for future studies.

**Table 1:** A summary of state-of-the-art in the literature.

*Papers*	*Text Language*	*Bug Reports Type*	*Classifiers*	*Ensemble scheme*	*Dataset (Type)*
[1]	English & Turkish	Unstructured	NB, SVM, KNN, LR, DT, and RF	No	Proprietary Dataset (Commerical)
[2]	English	Structured	Grid Serach	No	Eclipse, Apache, Mozilla (open source)
[4]	English	Structured	RF, NB, SVM, KNN, Bagging, Ada Boosting	Yes	Eclipse (open source)
[5]	English	Structured	MNB, LR, DT, and RF	No	Kaggle (open source)
[14]	English	Structured	NB, TAN and KBD	No	Compendium & Mozilla (open source)
[15]	English	Structured	DT, NB, and Bagging	Yes	Kaggle (open source)
[16]	English	Structured	LR, RF, SVM and LSTM	No	Mozilla (open source)
[17]	English	Structured	NB, LR, DT, and RF	No	Eclipse and Mozilla (open source)
[18]	English	Structured	MNB, SVM, LR, KNN, DT, XGB, NN, RF	No	Mozilla (open source)
[19]	English	Structured	SVM and NB	No	Eclipse and Mozilla (open source)
[20]	English	Structured	LR, RF, DT, SVM	No	Real Bug Dataset
[21]	English	Structured	RNN	No	Android APK files
***This Study***	***Persian***	***Unstructured***	***MNB, SVM, GNB*** ***LR and RF***	***Yes***	***Real Bug Dataset*** ***(Case study)***

## 2. Problem description

**Fig 1:**
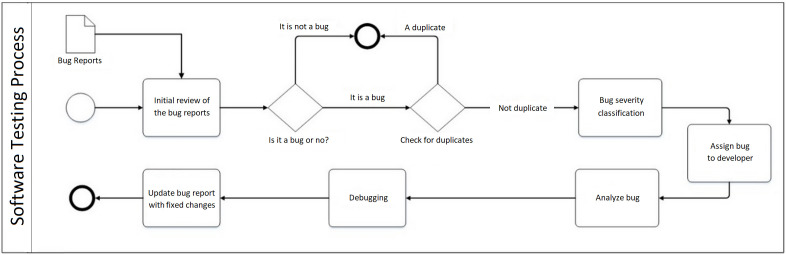
Software testing process flowchart.

The occurrence of bugs in the product development process is an inevitable fact. One of the most important activities before the software release is the implementation of a testing process to remove bugs in the final product. These bugs are recorded in writing by the test team or users in the form of bug reports. According to Fig 1, these bug reports are reviewed in the first stage of the testing process. In the initial review, reports that are not bugs or are duplicates are removed from the testing process and other reports are transferred to the classification stage. After determining the bug severity and classifying bug reports, the appropriate developer is selected and assigned to each bug report with a certain severity. The developers first analyze the roots of bugs, then debug them, and finally update the necessary changes to fix the software faults.

One of the most significant and time-consuming activities during the above-mentioned process is the bug severity classification which is usually done manually by experts in the traditional business environment. Since the manual implementation of this activity creates remarkable time and cost challenges for companies, many managers and researchers have recently tried to provide models to automate the bug severity classification using ML algorithms and text mining concepts. The bug report classification is necessary because firstly, it makes the team focus on the most important and risky bugs that have a stronger effect on the user experience of the product, and secondly, the testing process is facilitated by appropriately assigning the bug reports to the developers.

The format of recording bug reports may be different in various companies depending on the project, methodology and other factors. In general, these reports are presented in two formats, structured or unstructured. Unstructured bug reports contain free-text explanations of the bugs, while structured bug reports contain more detailed and specific information about the bug, such as the expertise domain of the bug. Since this detailed information about the specific features of bugs is not present in unstructured reports, the only way to extract this information is to mine their text and extract information from words. Therefore, this paper investigates the problem of automated bug severity classification in the Persian language using different multiclass ML methods. Also, it combines their predictions of these methods for the final classification using by an ensemble learning scheme based on voting concepts.

Bug severity is the impact level of a fault on the software system’s performance. This paper considers three degrees of bug severity with titles “Critical”, “Major”, and “Minor” for classifying the bug reports. Critical bugs are faults that block critical functions of software and cause a fundamental failure such that there is no way for the user to bypass these bugs. Major bugs are defects that can affect the functionality of an important module of the software so that the users encounter considerable challenges while working with the system. Finally, minor bugs are faults that usually do not cause serious failure in software functionality, so that the user can easily choose another way to avoid encountering these bugs.

According to what has been explained, in general, this paper distinguishes itself from other studies in a combination of the following features:

- ***Different ML-based techniques*:** The study applies various ML algorithms such as SVM, MNB, GNB, LR and RF for automated software bug report classification and compares their effectiveness over manual bug classification.
- ***Ensemble learning scheme*:** The paper uses an ensemble learning scheme based on the hard and soft voting approach for synthesizing the results of the mentioned ML algorithms to improve the efficiency of the bug classification model.
- ***Persian language bug reports*:** The investigated bug reports in this paper are written in Persian language which has a specific grammar syntax in comparison to other languages. Hence, tokenization activities are complex due to the existence of informal and colloquial words, and half-spaced words that are mistakenly typed without half-spaced in the Persian.
- ***Real dataset of bug report*s:** The paper utilizes a real dataset of bug reports which are extracted from the repository of bug reports related to a large-scale commercial software project in a case study.
- ***Unstructured bug reports*:** The research is involved with unstructured bug reports classification that has more complexity than structured bug reports classification due to the short text of unstructured bug reports which sometimes even finding the bug’s root cause for human intelligence is problematic. Thus, the text preprocessing step for suitable classification should be done with more effort.
- ***Bug report related to black-box testing*:** The used bug reports in this study have arisen from black-box testing such that the testers do not have access to source codes when filing bug reports. Hence, even if the bug report is described in detail, it might be required to check the source codes to correct the classification of the bug severity. Therefore, it is more difficult to classify the bug reports based on the text.

## 3. Research methodology

**Fig 2:**
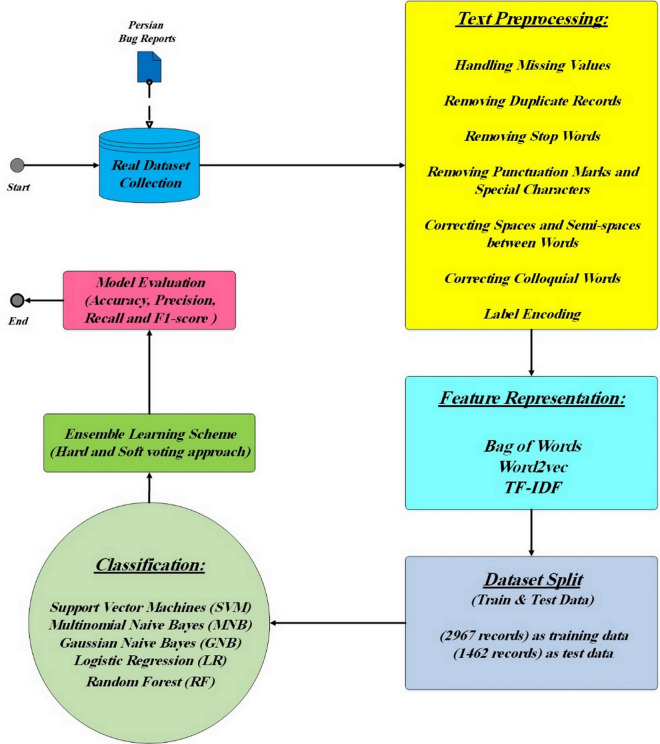
Problem solution process model.

This section describes the proposed solution for the automated bug severity classification problem. This paper to solve this problem, first collects data related to bug reports using bug tracking systems and other resources available in the company. Then, the text preprocessing package is performed as one of the most important steps of this solution. This package includes an integrated set of activities such as removing irrelevant information, managing missing values, removing stop words, managing spaces and half-spaces between words, colloquial words, etc. Next, the text features and labels related to the bug severity class (Critical, Major and Minor) are extracted and each of the bug reports is converted into a word representation vector so that ML algorithms can use them. Afterward, the labeled samples are divided into two groups of training and test data to use in the selected ML algorithms. In the next step, firstly, the selected machine learning algorithms, including SVM, MNB, GNB, LR and RF are trained to create a classification model. After training the classification model, its performance is evaluated on the test dataset. Then, the results of the mentioned ML algorithms are combined with an ensemble learning scheme based on hard and soft voting approaches. Finally, the performance of classifiers is assessed in terms of various metrics such as accuracy, precision, recall and f1-score to determine the effectiveness of the model. Moreover, the results of the proposed model for the classification of bug reports are compared with the results of classes determined based on elite opinion. The above process is briefly shown in Fig 2.

### 3.1. Dataset description

Data collection is the first and most basic step for data analysis, which is usually done using one of the following two approaches:

a) Using the gathered and published standard open-source data that are publicly available
b) Collecting real data from special case studies that are not publicly available.

Since there is no public and open source dataset related to bug reports in the Persian language, this study applies a real dataset containing Persian bug reports related to a special large-scale product. The records of this dataset have been compiled from the bug-tracking system and other information sources available in the company. This dataset includes two columns such that the text of bug reports is shown in the first column and the labels of their severity class in the second column with an integer number (Critical (1), Major (2) and Minor (3)). After preprocessing, the dataset contains 4,429 records, with 1,459 critical bugs, 1,583 major bugs, and 1,387 minor bugs.

Since the number of bug reports in each class is similar, the dataset does not require balancing. Also, one of the significant factors in obtaining accurate models in ML algorithms is having a large amount of training data. In this regard, this research randomly considers two-thirds of the records in the dataset (2967 records) as training data and one-third (1462 records) as test data.

### 3.2. Text preprocessing

Data preprocessing is a time-consuming and challenging step in classification problems that prepares data for model training. Also, it is one of the most significant steps of text mining, especially in Persian language texts, because the existence of special writing rules reduces the model accuracy if the Persian raw text is directly used to train the machine learning model. Text preprocessing is an integrated package of activities such as includes actions such as removing missing values, duplicate records, stop words, unwanted half-spaces/spaces, punctuation marks, numbers, special characters, foreign letters and correcting colloquial words, spelling mistakes, etc. It is obvious that the presence of each of the above-mentioned items in the text reduces the efficiency of a classification model. Therefore, to preprocess the Persian text in this paper, the above activities have been taken using the Hazm library in the Python software.

### 3.3. Feature representation

Generally, unstructured data such as texts must be converted into a structured feature space for use in classification models. Thus, feature extraction methods must be applied after text preprocessing. The study uses the Bag of Words (BoW), Term Frequency-Inverse Document Frequency (TF-IDF) and Word2vec approach in this regard.

The BoW is a common approach for converting text sentences into simplified numerical representations that considers a document or a sentence as a bag of words. In this method, the number of words in each document is counted without considering the semantic relationship between words. One of the most important drawbacks of BoW is not considering the word frequency which may change greatly from one report text to another. Hence, a prevalent statistical method called TF-IDF is used to overcome this problem. TF-IDF measures the importance level of a term within a bug report relative to a collection of bug reports by multiplying the word frequency by the inverse document frequency.

The BoW and TF-IDF methods do not consider the word semantics. For example, the words “defect”, “fault”, “flaw”, and “imperfection” are usually applied in the same context while the vectors related to these words are orthogonal in the BoW approach. This drawback leads to a significant issue in understanding sentences within the model. Consequently, this paper solves this problem by word embedding similar to many previous studies. Word embedding is a technique in which each word from the vocabulary is mapped to an N-dimension vector of real numbers to translate unigrams into understandable input for ML algorithms. Word2Vec is one of the most popular word embedding methods that have been successfully used in the literature. This approach creates low-dimensional data, unlike TF-IDF modeling.

This study considers each keyword extracted from the text as a feature in the data matrix. Since this paper used the Word2Vec method that groups synonymous words as similar features, it significantly reduces the statistical dependence between the extracted words. Moreover, we employed the chi-square method as a non-parametric feature selection approach to select appropriate features. This further minimizes redundancy among the final feature vectors and enhances the performance of machine learning algorithms. The chi-square method assesses the statistical dependence between features and classes by calculating Chi-squared scores. A higher score indicates a stronger association between a term and a class, highlighting its relevance for classification.

### 3.4. Classification model

After the preprocessing step and determining the training matrix of bug reports, supervised ML algorithms are used to construct the bug reports classifier models. The algorithms implement classification using the input matrix and learn the pattern of each class and its features. After being trained, an algorithm can classify new instances with unknown classes to the appropriate class.

Generally, there are different ML classifiers for bug severity classification. Hence, the proposed paper examines five of the most common ones consisting of SVM, Multinomial NB, Gaussian NB, multinomial LR and RF to evaluate their performance quality in multiclass bug report classification.

### 3.5. Ensemble learning scheme

The ensemble ML technique fuses several individual base models to enhance the predictive performance of the classification model [23]. Due to the functional diversity of various ML algorithms, the ensemble learning scheme can achieve better prediction than any of the learning models alone because the ensemble approach is performed by considering the results of all individual models and is not affected by misclassifications. In other words, in this approach, the powerful performance of other classifiers can compensate for the weakness of another classifier. The approach indicates the best performance when single models have sufficient variety and high prediction accuracy.

**Fig 3:**
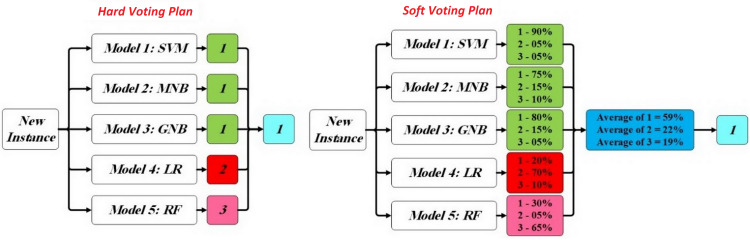
An numerical example of the plans of voting method.

Generally, “Bagging”, “Boosting” and “Voting” are three of the most common ensemble approaches in the literature [24]. The bagging method makes several models from different subsamples of the training dataset whereas the boosting method makes a framework that each model corrects the prediction errors of the previous model in a chain. In this paper, after developing the algorithms of SVM, MNB, GNB, LR and RF as single models, we apply the voting method for combining the five ML classifiers indicated above. This ensemble scheme is used to integrate predictions of single models by simple statistics. There are two prediction plans in the voting methods [24] that the Fig 3 provides an numerical example of them:

- *Hard Voting Plan:* This plan investigates the class labels predicted by each of the single models and selects one of them that has the most votes from these algorithms as the final class.
- *Soft Voting Plan:* This plan predicts the final class by the class label with the highest summed probability from the single models. Indeed, this plan involves the probability scores for each class label in the final decision.

## 4. Result model and evaluation

This section investigates the result of classification models for suggested case study. As already mentioned, five text classification algorithm which have been applied in this study with various vectorization methods and experimental setups are SVM, MNB, GNB, LR and RF. The hyperparameters of them are tunned by RandomizedSearchCV which is faster than GridSearchCV [25]. This approach tries to explore a wider range of hyperparameter values and efficiently investigates the model for each combination of the values by the Cross-Validation method.

To evaluate the performance of the classifiers, four metrics called accuracy, precision, recall, and F1-score are commonly applied. It is obvious that a high accuracy alone may not confirm proper performance of a algorithm if other metrics means the precision, recall, or F1-score are low. Thus, it is nessecary to evaluate all four mentioned metrics to investigate the overall performance of the model [26]. This study has been used sklearn library in Python to calculate the mentioned metrics for each of the classifiers.

The formula of accuracy, precision and recall are according to Table 2 [24]. For example, to calculate the precision in the multiclass classification problem, we first obtain the number of true and false positives and use the binary formula to determine the precision for each class individually (PRECk). Then, we calculate the average of PRECk for three classes (n = 3) by applying the multiclass formula. The same calculation procedure is used for accuracy and recall metrics. Eventually, the f1-score is calculated based on the values obtained for precision and recall in the multiclass classification problem.

**Table 2:** Metrics formula [24].

Metric	Formula	
	Binary classification	Multiclass classification
Accuracy	$ACC=\frac{TP+TN}{TP+TN+FP+FN}$	$ACC=\frac{\sum_{k=1}^{n}{\text{ACC}}_{k}}{k}$
Precision	$PREC=\frac{TP}{TP+FP}$	$PREC=\frac{\sum_{k=1}^{n}{\text{PREC}}_{k}}{k}$
Recall	$REC=\frac{TP}{TP+FN}$	$REC=\frac{\sum_{k=1}^{n}{\text{REC}}_{k}}{k}$
F1-score	$F1-score=2\times \frac{PREC\times REC}{PREC+REC}$	$F1-score=2\times \frac{PREC\times REC}{PREC+REC}$

Moreover, the mentioned algorithms are evaluated based on a common and powerful tool called receiver operating characteristics (ROC). The ROC curve illustrates the relationship between the true-positive rate (TPR/Sensitivity) and false-positive rate (FPR/1-Specificity). Also, the area under the ROC curve (AUC) assesses the performance of a classifier based on how well it can distinguish positive and negative classes with different thresholds. The TPR and FPR are formulated according to Equations (1) and (2), respectively.


$$TPR=\frac{TP}{TP+FN}=\text{Sensitivity}$$ (1)

$$FPR=\frac{FP}{FP+TN}=\text{1}-\text{Specificity}$$ (2)

The ROC is typically applied in binary classification problems where the TPR and FPR can be clearly determined. To utilize ROC in multi-class problems, the TPR and FPR values can be calculated only after binarizing the outputs. This can be implemented in two various ways:

1. The One-vs-Rest scheme that compares each class against all the others.
2. The One-vs-One scheme that compares every unique pairwise combination of classes.

This paper uses the first scheme for depicting the multiclass ROC curve.

This section analyzes the results of the automated bug severity classification process in different sub-sections. First, Subsections (4.1) – (4.5) express the results of the automated software bug severity classification by SVM, MNB, GNB, LR and RF algorithms, respectively. Also, Subsection (4.6) indicates the results of the ensemble learning scheme in this study. Finally, a comparative study is given in Subsection (4.7) to demonstrate the performance of the five classifiers mentioned and the voting method compared to each other.

### 4.1. Results of SVM algorithm

This section presents the results of the execution of the automated bug severity classification process for the provided case study by the SVM algorithm. According to Table 3, the SVM algorithm with 91.45% accuracy has shown a very good performance in bug severity classification. Also, the values of precision, recall and F1-score for the three existing classes are 89% to 95%, 88% to 98% and 89% to 97% respectively. These results illustrate that the SVM has an average score between 91 and 92 percent in all four mentioned metrics, which indicates the high reliability of this algorithm for classifying the software bug severity.

**Table 3:** The results of bug reports classification by the SVM algorithm.

	Precision	Recall	F1-score	Support
Class 1	0.90	0.89	0.90	489
Class 2	0.89	0.88	0.89	526
Class 3	0.95	0.98	0.97	447
Accuracy	91.45%			1462
Macro Average	0.92	0.92	0.92	1462
Weighted Average	0.91	0.91	0.91	1462

Another noteworthy point about the above results is that values of precision, recall and F1-score in the second class (Major bugs) are a little less than these values in the first (Critical bugs) and third (Minor bugs) classes. This challenge exists even in manual classification by experts and human intelligence because the severity of the bugs that are classified in the second class (Major) is close to the severity level of critical or minor bugs in some cases, hence it causes difficulty for software testing experts in determining the proper severity level of these bugs.

**Fig 4:**
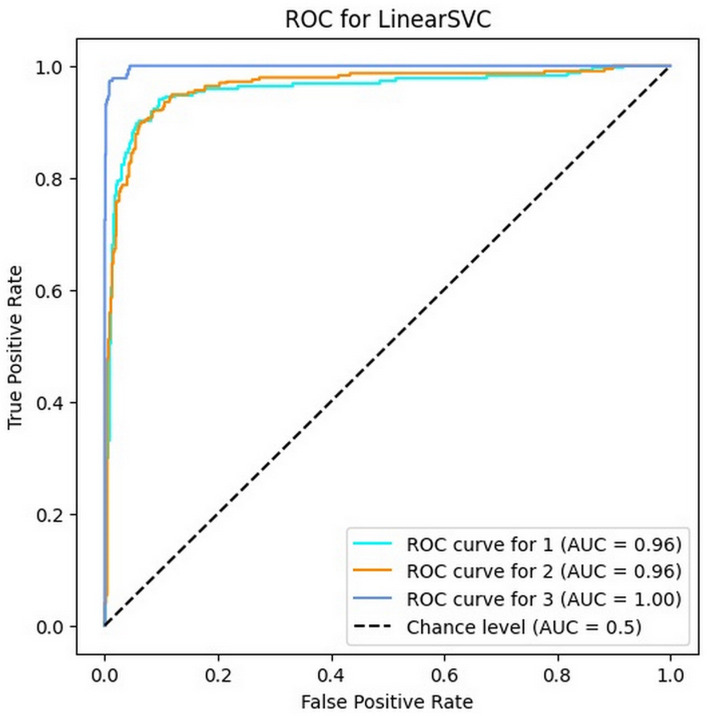
The ROC of SVM for three classes.

Fig 4 shows the ROC of the SVM classifier for three different classes. As it is clear, the AUC values for all classes are more than 0.95, which indicates the excellent efficiency of this algorithm in classifying the severity of software bug reports.

### 4.2. Results of MNB algorithm

In this section, Table 4 demonstrates the results of automated bug severity classification by the MNB algorithm. According to these results, the accuracy of the MNB algorithm is equal to 82.9% ≈ 83% and the values of precision, recall and F1-score are [76–93]%, [78–87]% and [80–90]%, respectively. The results indicate that this algorithm has acceptable performance for classifying Persian bug reports, but it cannot be used with a high confidence factor in bug severity classification.

**Table 4:** The results of bug reports classification by the MNB algorithm.

	Precision	Recall	F1-score	Support
Class 1	0.83	0.78	0.80	489
Class 2	0.76	0.84	0.80	526
Class 3	0.93	0.87	0.90	447
Accuracy	82.9%			1462
Macro Average	0.84	0.83	0.83	1462
Weighted Average	0.83	0.83	0.83	1462

Among the four evaluated metrics, the recall has a worse situation than the others, especially in the first (critical) and third (minor) classes. This confirms that the MNB algorithm has a high false negative, which means that it mistakenly predicts the critical or minor classes for bug reports that are not in these categories. Since this problem can lead to an additional cost to the software company, this weakness should be taken into account if this algorithm is used for automated bug severity classification.

**Fig 5:**
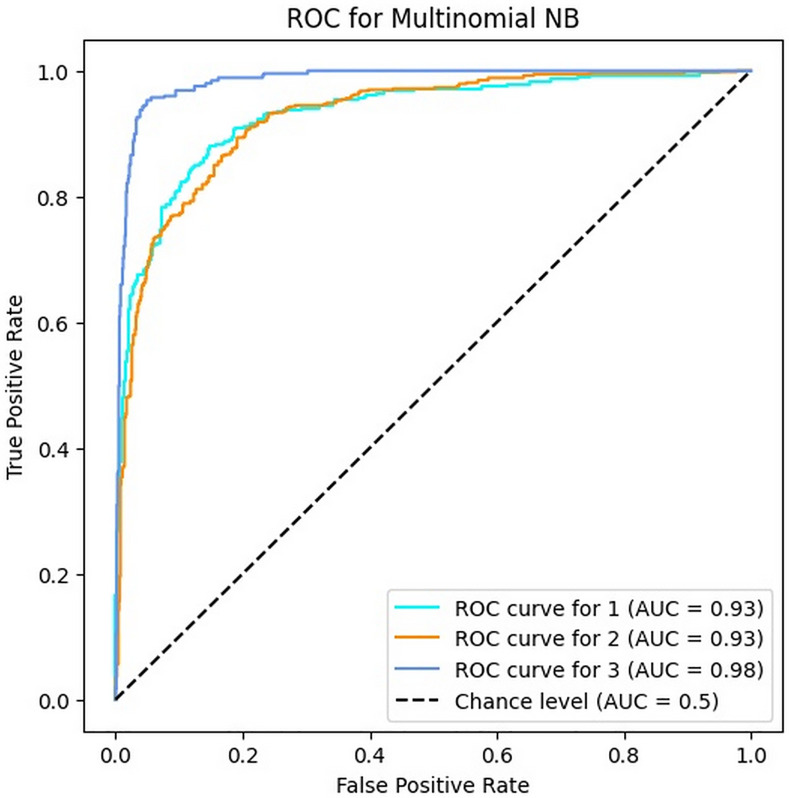
The ROC of MNB for three classes.

Fig 5 displays the ROC curves of MNB for three classes in which the AUC values for classes one, two and three are 0.93, 0.93 and 0.98, respectively. These results confirm that the MNB classifier has a very good performance with a slight difference compared to the SVM algorithm.

### 4.3. Results of GNB algorithm

This section depicts the results of the implementation of the bug severity classification by the GNB classifier in Table 5. The results confirm this algorithm similar to SVM has very good performance in Persian bug classification such that its accuracy is about 90% (exactly 89.81%) and the values of precision, recall and F1-score are [86–96]%, [86–98]% and [87–97]%, respectively. These results denote that the GNB is about 90% percent on average in all four metrics, which means that this algorithm can be useful for classifying the severity of Persian software bugs.

**Table 5:** The results of bug reports classification by the GNB algorithm.

	Precision	Recall	F1-score	Support
Class 1	0.88	0.86	0.87	489
Class 2	0.86	0.87	0.87	526
Class 3	0.96	0.98	0.97	447
Accuracy	89.81%			1462
Macro Average	0.90	0.90	0.90	1462
Weighted Average	0.90	0.90	0.90	1462

Another remarkable point about the results is that values of precision, recall and F1-score are better in the third class (Minor bugs) than these values in the first and second classes (Critical and Major bugs). As mentioned in Section 4.1, this issue sometimes occurs even in the manual classification of bugs by experts because the exact separation of critical and major severity levels is very difficult for some bug reports.

**Fig 6:**
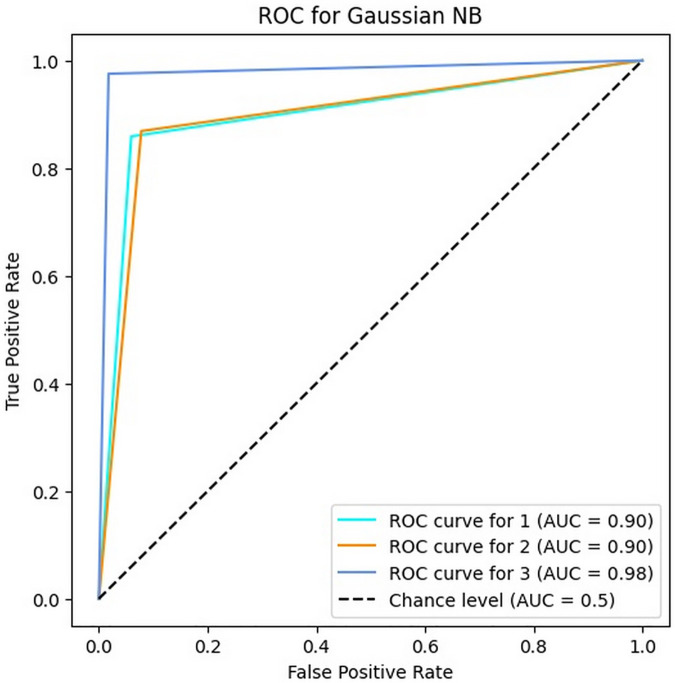
The ROC of GNB for three classes.

Fig 6 demonstrates the ROC of the GNB classifier for three classes. According to it, the AUC values for classes one, two and three are 0.90, 0.90 and 0.98, respectively. These results confirm that the GNB classifier has a very good performance similar to the MNB classifier.

### 4.4. Results of LR algorithm

In this section, the results of the LR algorithm in bug reports classification are provided in Table 6. The results show the accuracy, precision, recall and F1-score of the LR algorithm are 86.39%, 81% to 92%, 80% to 96% and 83% to 94% respectively. Therefore, LR similar to MNB has an acceptable performance for bug report classification, but it cannot be utilized with a high reliability in bug severity classification. Moreover, the values of precision, recall and F1-score of this algorithm as same as GNB are lower in the first and second classes (Minor and Major bugs) in comparison to these values in the third class (Critical bugs).

**Table 6:** The results of bug reports classification by the LR algorithm.

	Precision	Recall	F1-score	Support
Class 1	0.87	0.80	0.83	489
Class 2	0.81	0.84	0.83	526
Class 3	0.92	0.96	0.94	447
Accuracy	86.39%			1462
Macro Average	0.87	0.87	0.87	1462
Weighted Average	0.86	0.86	0.86	1462

**Fig 7:**
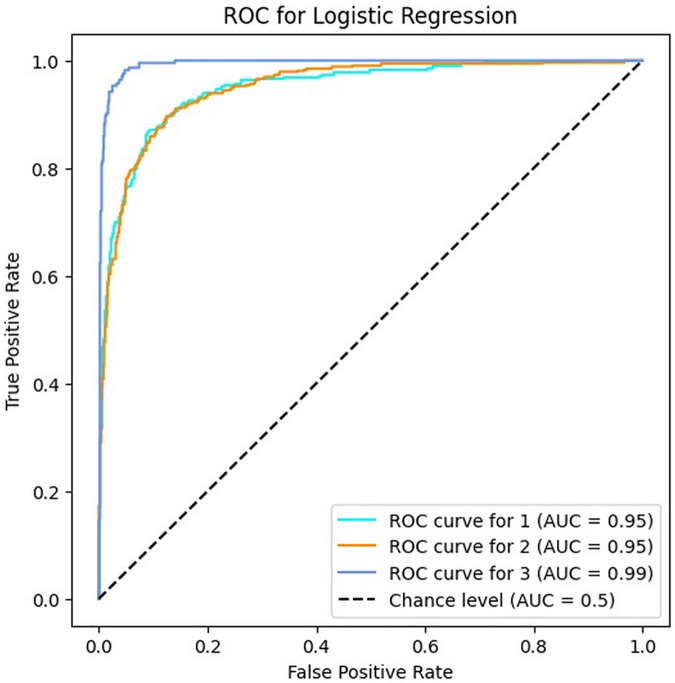
The ROC of LR for three classes.

Fig 7 indicates the ROC curves of the LR algorithm for three classes. According to it, the AUC values for all classes are very close to their corresponding values in the SVM algorithm. These results denote the high power of the LR algorithm in predicting the severity of bugs.

### 4.5. Results of RF algorithm

**Fig 8:**
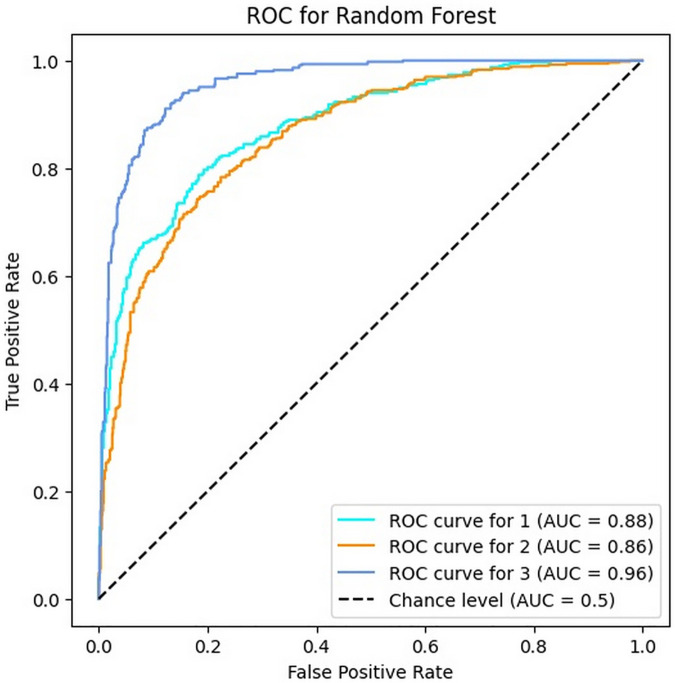
The ROC of RF for three classes.

This section displays the results of the RF algorithm for bug severity classification in the investigated case study. According to Table 7, the RF with 65.05% accuracy has shown an unreliable performance for solving the problem of bug reports classification. Indeed, although this algorithm has been relatively able to classify Persian software bugs, this level of accuracy cannot be used instead of human intelligence because the costs of misclassification by this algorithm will be relatively high for software companies. Additionally, the values of precision, recall and F1-score demonstrate random forest low reliability for bug severity classification in comparison to manual classification. On the other hand, Fig 8 illustrates the RF algorithm has lower performance in comparison to other classifiers in terms of the AUC value, too.

**Table 7:** The results of bug reports classification by the RF algorithm.

	Precision	Recall	F1-score	Support
Class 1	0.89	0.48	0.63	489
Class 2	0.51	0.93	0.66	526
Class 3	0.93	0.51	0.66	447
Accuracy	65.05%			1462
Macro Average	0.78	0.64	0.65	1462
Weighted Average	0.77	0.65	0.65	1462

### 4.6. Results of ensemble learning scheme

After using the ensemble learning scheme by hard and soft voting plans on the predicted results of SVM, MNB, GNB, LR and RF as basic models, remarkable results were obtained and the summary of them is shown in Table 8.

**Table 8:** The results of bug reports classification by the voting approach.

Voting Plan	Class	Precision	Recall	F1-score	Support
Hard voting	1	0.94	0.90	0.92	489
	2	0.90	0.93	0.92	526
	3	0.97	0.98	0.98	447
	Accuracy	93.64%			1462
	Macro Average	0.94	0.94	0.94	1462
	Weighted Average	0.93	0.94	0.94	1462
Soft voting	1	0.99	0.93	0.96	489
	2	0.93	0.98	0.95	526
	3	0.98	0.99	0.99	447
	Accuracy	96.51%			1462
	Macro Average	0.97	0.97	0.97	1462
	Weighted Average	0.97	0.97	0.97	1462

The results of the above table show that the use of an ensemble learning scheme leads to a significant improvement in the performance of the classification model. As it has been indicated, the accuracy of the model is 93.64% and 96.51% for hard and soft voting plans, respectively. According to this level of accuracy and low prediction error, using this approach to automatically classify the severity of software bugs will be very cost-effective. In addition, the values of precision, recall and F1-score demonstrate the very good performance of these approaches. Among the two plans of hard and soft voting, the soft voting plan presents a better performance in all four metrics.

### 4.7. The comparative study of classifiers

**Fig 9:**
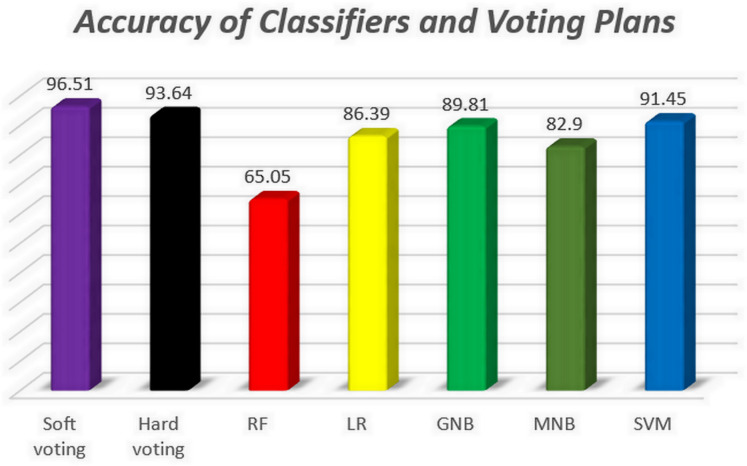
The accuracy of classifiers and voting plans.

In this section, to compare the performance of the classifiers evaluated in this study, the results of the automated bug severity classification by these algorithms have been examined based on the values of accuracy, precision, recall and F1-score. For this purpose, the first, the accuracy of five classifiers and voting plans is illustrated in Fig 9. Afterward, the macro average and weighted average of precision, recall and F1-score for all classifiers and voting plans are displayed in –, respectively.

**Fig 10:**
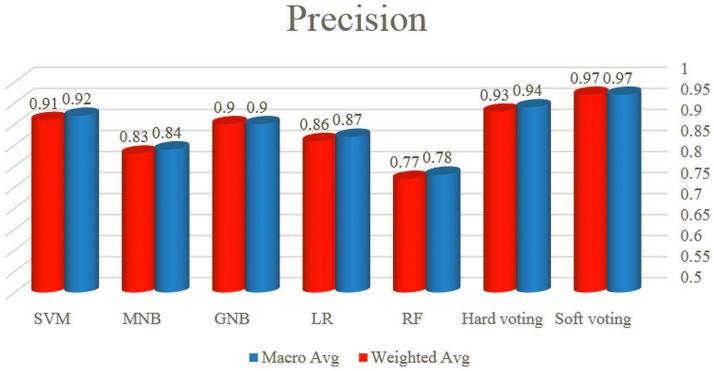
The macro and weighted average of precision for all classifiers and voting plans.

**Fig 11:**
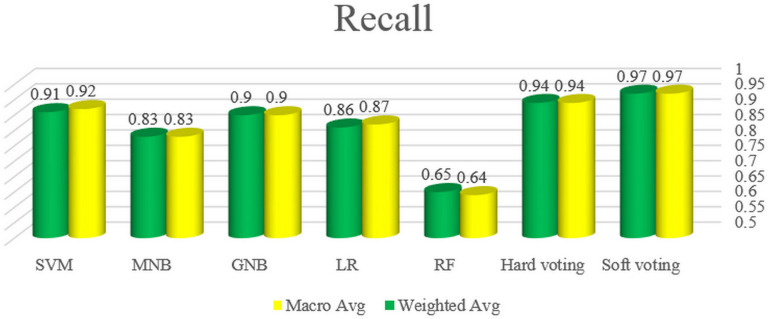
The macro and weighted average of recall for all classifiers and voting plans.

**Fig 12:**
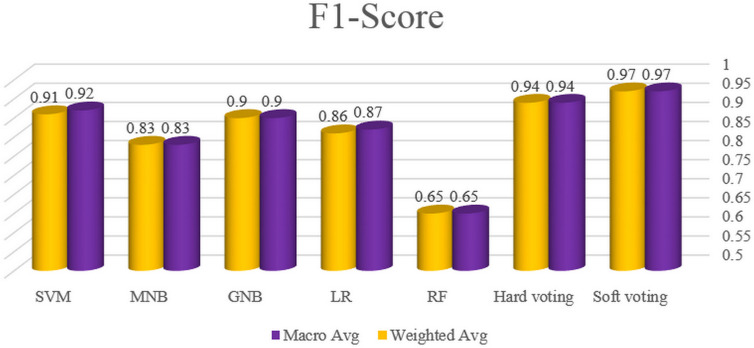
The macro and weighted average of F1-score for all classifiers and voting plans.

Fig 9 demonstrates that in terms of accuracy measure, the SVM algorithm with 91.45% has the best performance in automatically classifying the severity of bug reports among five single algorithms, followed by the GNB and LR algorithms with 89.81% and 86.39% accuracy ranked second and third. Moreover, the RF algorithm shows the worst performance in classification. Also, as expected, ensemble approaches have higher accuracy than all five basic models and the soft voting plan is more accurate than the hard voting due to the more appropriate voting procedure.

In general, the graphs of the macro and weighted average of precision, recall and F1-score measures confirm that the best performance among five single algorithms in software bug classification is related to SVM, GNB, LR, MNB and RF, respectively. Albeit, due to the closeness of the results of SVM and GNB algorithms, it can be said that both algorithms are suitable and reliable for classifying the bug severity in the Persian language. Furthermore, hard and soft voting respectively have values of 94% and 97% in precision, recall and f1-score and both operate better than all single models based on the mentioned metrics.

In the following, the details of the results related to precision, recall and F1-score for the evaluated algorithms are fully shown in Table 9.

**Table 9:** The details of the results related to the evaluated algorithms.

Metrics	Algorithm	Classes			Averages	
		1 Critical Bugs	2 Major Bugs	3 Minor Bugs	Macro	Weighted
Precision	SVM	0.90	0.89	0.95	0.92	0.91
	GNB	0.88	0.86	0.96	0.90	0.90
	LR	0.87	0.81	0.92	0.87	0.86
	MNB	0.83	0.76	0.93	0.84	0.83
	RF	0.89	0.51	0.93	0.78	0.77
	Hard voting	0.94	0.90	0.97	0.94	0.93
	Soft voting	0.99	0.93	0.98	0.97	0.97
Recall	SVM	0.89	0.88	0.98	0.92	0.91
	GNB	0.86	0.87	0.98	0.90	0.90
	LR	0.80	0.84	0.96	0.87	0.86
	MNB	0.78	0.84	0.87	0.83	0.83
	RF	0.48	0.93	0.51	0.64	0.65
	Hard voting	0.90	0.93	0.98	0.94	0.94
	Soft voting	0.93	0.98	0.99	0.97	0.97
F1-score	SVM	0.90	0.89	0.97	0.92	0.91
	GNB	0.87	0.87	0.97	0.90	0.90
	LR	0.80	0.84	0.96	0.87	0.86
	MNB	0.80	0.80	0.90	0.83	0.83
	RF	0.48	0.93	0.51	0.65	0.65
	Hard voting	0.92	0.92	0.98	0.94	0.94
	Soft voting	0.96	0.95	0.99	0.97	0.97

## 5. Discussion

In this section, we compare the top three single algorithms of this study from another aspect. It is clear that any mistake in the automated bug severity classification can lead to the imposition of costs on software companies; But due to the higher cost of occurrence and debugging of critical bugs compared to major and minor bugs, if critical bugs (first class) are wrongly predicted in the second or third classes, the cost imposed can be much higher. Therefore, although SVM, GNB and LR algorithms have less misclassification rate in prediction, it is the superior algorithm that predicts critical bugs more accurately. The results of the precision, recall and F1-score criteria in the first class indicate that the SVM algorithm has a better performance than GNB and LR in this aspect among five single models. More precisely, the results show that the SVM algorithm mistakenly detected 48 critical bugs as minor bugs, but this value is equal to 58 and 57 for the GNB and LR algorithms, respectively.

According to the above description can be said that the recall is most important metric in this problem because a false negative has a higher cost than a false positive, particularly in classes one and three. The results indicate that SVM and GNB have the best performance in terms of recall measure among single classifiers. Moreover, the ensemble approach based on the soft voting plan illustrates excellent performance in this metric in comparison to other classifiers.

Generally, the present study describes that text mining and ensemble methods based on voting can significantly reduce the time of bug severity classification compared to manual classification by humans while having very good accuracy in bug classification.

In addition to the findings presented in the results section, we performed practical evaluations to test real-world scenarios and assess the model’s performance compared to the company’s current methods. Specifically, we observed five meetings focused on analyzing and classifying software bug reports. Typically, the attendees at these meetings consist of the product manager, the manager of the development team, a senior developer, as well as the manager and senior expert from the testing team. During these observations, we recorded the duration of each meeting, the number of participants, the total number of classified bug reports, and the classification categories assigned to each report. A total of 92 software bugs were classified across these five meetings.

Subsequently, we deployed the proposed model within the company, integrating it with the bug tracking system. The text of the same 92 bug reports reviewed by experts during the meetings was reclassified using the model. We also recorded the time required for the model’s installation, training, and classification processes.

Our observations revealed that only one-third of the total meeting time was dedicated to classifying the severity of bug reports, while the remainder was spent on other discussions. Therefore, for a fair comparison, only one-third of the total time from the five meetings was considered for bug severity classification.

The comparative results demonstrated that the proposed model could classify the severity of 92 bug reports in just one-sixth of the time typically required by traditional methods that do not utilize an automated model. Given that approximately 25 such meetings are held annually, involving experts and key specialists from the development and testing teams, the potential cost savings are substantial. With the high salaries of these professionals, reducing meeting durations can significantly lower the company’s expenses. According to the company’s financial experts, implementing this model could reduce costs associated with bug severity classification by 80%. Furthermore, the overall human resource expenditure during the software testing process could be decreased by 20%.

However, the real-world implementation of the model presented several challenges. These included resistance from managers and experts to change existing workflows due to ingrained work culture, initial skepticism regarding the model’s results, and technical issues in integrating the model with the bug tracking system. Despite these hurdles, the model’s high accuracy and significant time savings gradually fostered trust and collaboration, leading to its successful adoption and use.

## 6. Threats

This section describes some factors that may threaten the validity of the suggested research in three parts: (1) Construct threats, (2) Internal threats, and (3) external threats.

- *Construct validity*: We are aware of potential threats to the construct validity due to the choice of evaluation metrics. Therefore, we utilize commonly used metrics within the research community, such as accuracy, precision, recall, and F1-score, which have been employed by numerous researchers in sources [15,17], and [18]. Additionally, we incorporate the ROC to provide a more extensive model evaluation. However, it is obvious that an overreliance on the indicated metrics may pose a threat to construct validity. Furthermore, the choice of parameter values in classification algorithms can introduce additional validity concerns. To address this issue, our paper uses RandomizedSearchCV for parameter tuning that explores a broader spectrum of hyper-parameter values and analyzes the model for each combination of the values by the Cross-Validation method. This approach enables us to implement experiments aimed at identifying optimal parameter settings rather than relying on default values. However, it is essential to acknowledge that the selected algorithm parameters can significantly impact the results.
- *Internal validity*: This paper assumes that bug reports consist solely of text and do not include any attached images. In reality, some bug reports do contain images, which could enhance the accuracy of classification through image processing. Since a significant number of the bug reports in the examined dataset lacked attached images, this study focused only on the text of the bug reports. In the future, we plan to further investigate the influence of joint analysis of both the text and images of bug reports, provided we can obtain a suitable dataset.
- *External validity*: A threat to external validity is the generalizability of our results. We focus on the bug reports from a special product in the case study for the evaluation of the proposed approach because there is no public open-source repository for software error reports in Persian. The results of the proposed approach may not be consistent with bug reports from other projects. Whether our approach is feasible for different datasets in software projects should be analyzed by collecting other Persian bug report datasets.

## 7. Conclusion and future research

The problem of automated bug severity classification is an important challenge in actual work environments that has been studied by many researchers in recent years. This study provided an integrated approach of text mining, natural processing language and machine learning algorithms to improve previous studies and solve this problem for Persian bug reports. For this purpose, the paper applied various ML techniques such as SVM, MNB, GNB, LR and RF for multiple classifying of the severity of unstructured bug reports in the Persian language text. Also, it combines their predictions of single ML algorithms by ensemble learning scheme using two forms of voting plans. After collecting data from a real case study and special pre-processing of Persian text, each of the above algorithms was trained and finally, their performance was evaluated according to four metrics consisting of accuracy, precision, recall and f1-score. The results confirmed that the SVM, GNB and LR algorithms respectively are the best classifiers among mentioned single models for automatically classifying the severity of bug reports in terms of all mentioned metrics. Among these three algorithms, the SVM showed the best performance with 91.45% accuracy and an average of precision, recall and f1-score of about 91% to 92%. Moreover, this classifier performed better and more accurately than other algorithms for predicting the severity of critical bugs (class one). This advantage is remarkable because the incorrect classification of this class of bugs usually imposes more cost on the software development company compared to other classes. On the other hand, the results demonstrate that the ensemble method leads to a remarkable improvement in classification metrics in comparison to single models. Also, the soft voting plan indicates better performance in all evaluated metrics in comparison to other classifiers.

Generally, the present study offers practical insights for senior and executive managers of software development companies seeking to use data-driven strategies to automate time-consuming and costly software project activities. The first insight is the broad conclusion that using text-based approaches to automate activities can lead to better resource management, higher productivity of human capital, and deeper business analytics. The second managerial insight is that due to the high importance of the software testing process, managers in the field of product quality engineering should adopt new technologies based on artificial intelligence because such technologies reduce the time spent on time-consuming activities and provide useful insights about users’ opinions on the product. These insights can be used to prioritize tasks, optimally design the structure of software products, and debug the software bugs that have been reported.

To extend this paper as future research, we present the following suggestions:

- More accurate models for the classification of Persian bug severity should be presented by providing more accurate approaches in the pre-processing stage and other classifiers.
- In addition to using the text of bug reports, a new model should be developed for classifying software bugs based on images attached to bug reports.
- Moreover, the performance of machine learning algorithms should be evaluated for the classification of Persian bugs based on bug type including UI bugs, security bugs, coding bugs, etc.
